# Apolipoprotein E: Essential Catalyst of the Alzheimer Amyloid Cascade

**DOI:** 10.1155/2012/489428

**Published:** 2012-07-15

**Authors:** Huntington Potter, Thomas Wisniewski

**Affiliations:** ^1^Department of Molecular Medicine, Suncoast Gerontology Center, Johnnie B. Byrd Sr. Alzheimer's Institute, University of South Florida, Tampa, FL 33620, USA; ^2^Department of Neurology and Linda Crnic Institute for Downs Syndrome, School of Medicine, University of Colorado Aurora, CO 80045, USA; ^3^Departments of Neurology, Pathology, and Psychiatry, New York University School of Medicine, New York, NY 10016, USA

## Abstract

The amyloid cascade hypothesis remains a robust model of AD neurodegeneration. However, amyloid deposits contain proteins besides A**β**, such as apolipoprotein E (apoE). Inheritance of the apoE4 allele is the strongest genetic risk factor for late-onset AD. However, there is no consensus on how different apoE isotypes contribute to AD pathogenesis. It has been hypothesized that apoE and apoE4 in particular is an amyloid catalyst or “pathological chaperone”. Alternatively it has been posited that apoE regulates A**β** clearance, with apoE4 been worse at this function compared to apoE3. These views seem fundamentally opposed. The former would indicate that removing apoE will reduce AD pathology, while the latter suggests increasing brain ApoE levels may be beneficial. Here we consider the scientific basis of these different models of apoE function and suggest that these seemingly opposing views can be reconciled. The optimal therapeutic target may be to inhibit the interaction of apoE with A**β** rather than altering apoE levels. Such an approach will not have detrimental effects on the many beneficial roles apoE plays in neurobiology. Furthermore, other A**β** binding proteins, including ACT and apo J can inhibit or promote A**β** oligomerization/polymerization depending on conditions and might be manipulated to effect AD treatment.

## 1. Introduction

Alzheimer's disease (AD) is a neurodegenerative disorder that is clinically characterized by progressive mental decline and histopathologically defined by highly abundant amyloid deposits and neurofibrillary tangles in the brain parenchyma. The identification of mutations within the amyloid precursor protein (APP) and presenilin (PS) genes that cause autosomal dominantly inherited AD and that result in increased production of amyloid-prone forms of A*β* established beyond doubt that the processing of APP and the production of A*β* peptides are intimately involved in the disease process and led to the proposal and the reinforcement of the Alzheimer Amyloid Cascade Hypothesis [1, 2].

The role of amyloid in neuronal dysfunction has recently been extended by the discovery of small, soluble, oligomers of the A*β* peptide, some forms of which have been termed ADDLs (A*β*-derived diffusible ligands), protofibrils, or Aβ*56 [3–6]. These A*β* oligomers are not only potential intermediates in the formation of amyloid filaments, but they also have been shown to be neurotoxic themselves and to inhibit long-term potentiation (LTP), a cellular model of memory, in hippocampal slices [4, 7, 8]. Thus, the Amyloid Cascade Hypothesis now includes the essential role of A*β* oligomers in the neurodegeneration process.

 Despite its strength, the Amyloid Cascade Hypothesis is incomplete without including the essential role of amyloid-associated inflammatory proteins. For example, biochemical and histological studies first showed that, in addition to A*β*, amyloid deposits also contained the inflammation/acute phase protein *α*1-antichymotrypsin (ACT) [9] and, later, apolipoprotein E (apoE) [10, 11], which were both hypothesized to serve as catalysts or “pathological chaperones” of amyloid formation [9, 11, 12]. These and other results also indicated that Alzheimer's disease and its manifestation in middle-aged Down syndrome may include an inflammatory process, for both ACT and apoE are inflammatory and/or acute phase proteins in other contexts, and both are overexpressed in affected regions of the AD brain (for reviews see [13–15]). Indeed, Alzheimer himself first identified the inflammatory component of Alzheimer's disease when he described reactive astrocytes and microglia in affected brain regions of his first patient [16]. However, until inflammatory proteins such as ACT, IL-1, HLA, and apoE were found to be overexpressed in AD and DS brains, the term “inflammation” was explicitly excluded from the clinical and pathological description of AD because of the lack of edema and lymphocyte infiltration [9–11, 17, 18].

The significance of these biochemical results instigated and was reinforced by parallel genetic discoveries implicating a role for inflammation in AD. In particular, inheritance of the apoE *ε*4 allele was found to be the strongest known risk factor for AD besides age, with one copy increasing AD risk 3–5-fold and two copies over 10-fold [19–21]. Furthermore, apoE *ε*4 promotes cognitive decline in middle-aged Down syndrome individuals [22].

Because of apoE's essential genetic, and therefore presumably biochemical, contribution to AD pathology and cognitive decline, it is critical that its role in the AD pathogenic pathway/amyloid cascade be elucidated in order for therapeutics based on apoE to be designed. While recent excellent and encyclopedic literature reviews describe the many potential roles that apoE plays in AD [23–26], this focused review will concentrate on the interaction between A*β* and apoE and other inflammatory proteins, on the effects of such interactions, and on their implications for designing apoE-based AD therapies. The central question we try to answer is whether increasing or decreasing apoE level and/or function will serve best to reduce AD/DS pathology and cognitive decline. Lack of a clear answer may lead to the development of drugs that, rather than serving as an AD therapy, instead potentially exacerbate the disease.

## 2. Background: ApoE as Amyloid Catalyst

To determine whether inflammation contributes to Alzheimer's disease rather than being merely a correlative pathological feature in the AD brain, we and others tested the hypothesis that ACT and/or apoE serve as amyloid catalysts or pathological chaperones. Numerous *in vitro* and *in vivo* studies showed that mature amyloid deposition and the associated cognitive decline is strongly stimulated by apoE and ACT in a dose-dependent and isoform-specific manner, with apoE4 being the strongest promoter of A*β* polymerization and apoE2 being an inhibitor, paralleling the effect of these two isoforms in humans [27–38]. Indeed, without one or the other of these amyloid catalysts expressed in the brain, amyloid deposition is profoundly delayed in APP transgenic mice and does not become filamentous. Such APP+/apoE KO animals also exhibit normal cognition despite levels of A*β* expression equal to the apoE-expressing APP animals. Elegant work by Manelli and colleagues also showed that native lipidated apoE4 from transgene replacement astrocytes increases A*β* neurotoxicity compared to apoE3 or E2, indicating that apoE4 provides a negative gain of function [39]. Finally, Jones and colleagues recently showed that apoE4 also promotes the conversion and enhanced synaptic localization of A*β* as oligomers, the most neurotoxic form of the Alzheimer amyloid peptide [40, 41]. These recent studies extended prior work showing that apoE copurifies with A*β* during biochemical isolation of amyloid from human brains, and that apoE preferentially interacts with A*β* peptides in a *β*-sheet structure [42–45].

Together these results show that inflammatory proteins, particularly apoE, are integral parts of the amyloid cascade, and that without them the cascade would be arrested at the level of the harmless A*β* monomer, and no AD would ensue.

## 3. Background: ApoE in A*β*/Amyloid Clearance

The view of apoE as an integral and pathological part of the amyloid cascade has been shaken by experiments that suggest that apoE, far from being an amyloid catalyst, serves to clear A*β* from the brain. Under this view, ApoE is protective, with human apoE4 being less protective than apoE3 or E2 (for the most recent discussion, see [46] and commentary at http://www.alzforum.com/).

The first experiments that suggested apoE's role as a neuroprotector examined the pathology and cognition of APP transgenic mice carrying a second transgene expressing one or another human apoE isoform. Contrary to expectations, amyloid deposition in these mice was inhibited by the human apoE transgene, as though human apoE was protective [47]. Ultimately, the mice did develop amyloid, with the apoE4-expressing strain accumulating earlier and more extensive pathology [33, 34, 48, 49]. It was proposed that human apoE might serve to inhibit A*β* clearance from the brain compared to mouse apoE, with apoE4 inhibiting clearance the most. Other experiments showed that indeed, clearance of A*β* species was inhibited by complexing with apoE, especially apoE4 [46, 50].

The possibility that interaction with apoE modulated an A*β* clearance mechanism appeared to be supported by the finding that introduction of anti-A*β* antibodies or other A*β*-binding proteins such as gelsolin, led to a reduced amyloid load in the brain and rapidly improved cognition, with little evidence of A*β*-binding agents invading the brain parenchyma [41–54]. We also introduced apoE itself into the circulation via parabiosis and found that it induced amyloid clearance without entering the brain in AD model mice [38]. Thus the “Peripheral Sink Hypothesis” became a viable alternative or addition to the Amyloid Cascade Hypothesis, with apoE potentially playing an additional role as an A*β*-binding peripheral protein.

Most recently, an approach to therapy has been investigated in AD mice that is based on activating the liver X receptor (LXR), which also exists on other cells including microglia [55–57]. Activation of LXR results in increased expression of many proteins including apoE and its lipidating enzyme, ATP-binding Cassette Transporter A1 (ABCA1). The results indicate that activating LXR with the ligand GW3965 or the FDA-approved antiskin cancer drug bexarotene reduces soluble and insoluble A*β* and improves cognition in APP Tg mice, while knocking out the ABCA1 gene in APP mice showed a tendency to reduced amyloid load. Because apoE expression and lipidation is stimulated by LXR activation, the results were interpreted as proof that increased apoE levels help microglia clear A*β* and amyloid, as indeed some earlier cell culture experiments had suggested. However, it has also been shown that genetic overexpression of ABCA1 reduces amyloid deposition in mice where the apoE levels are unchanged [58]. Hence, because LXR stimulation influences the levels of many proteins, it is problematic to definitively link its *in vivo* action to the altered level of one particular protein. Furthermore, the increased levels of ABCA1 induced by Bexarotene enhance apoE lipidation, a change that is known to alter apoE/A*β* interactions. Hence, it is important to consider the lipidation state of apoE, which affects its function, in addition to the absolute levels of apoE.

## 4. Synthesis

 When trying to distinguish and weigh the value of two hypotheses, it is instructive to consider their testable predictions. If apoE is an amyloid catalyst, then reducing apoE levels or function in the brain should result in reduced amyloid deposition and reduced cognitive decline. If on the other hand, apoE is involved in A*β* clearance with human apoE4 being a greater inhibitor of clearance (or poorer clearer), then reducing apoE levels or apoE binding to A*β* should increase amyloid deposition and cognitive decline.

 All experiments carried out so far *in vitro* or in transgenic mice indicate that the ability of A*β* to form neurotoxic filaments or oligomers and cause cognitive decline are increased in the presence of apoE, particularly mouse apoE and human apoE4, with apoE2 being protective. In contrast, in the complete absence of apoE, the mutant APP gene and its product A*β* are harmless, generating neither amyloid deposits, synaptic disfunction, or cognitive decline, with one copy of apoE having an intermediate effect, as discussed above. The *in vitro* experiments in particular indicate that apoE likely acts catalytically to promote A*β* polymerization, as the molar ratio of A*β* to apoE of about 200/1 was appropriate for the formation of neurotoxic products [27–30]. Most recently, earlier work showing that mice expressing only one apoE gene accumulated less amyloid than those with two apoE genes (32) was repeated in two different laboratories using human apoE knock-in mice, and the same result was found, that is, lower doses of apoE3 or apoE4 led to reduced amyloid deposition [59, 60].

 The simplest interpretation of the *in vitro*, cell culture, and transgenic mouse data is that apoE is necessary for A*β* to polymerize into neurotoxic oliogomers/filaments, probably by binding to A*β* and thus altering its structure more toward the *β*-sheet and more easily allowing successive A*β* peptides to add on to the growing chain. The recent finding that apoE promotes A*β* oligomer formation *in vivo* reinforces this interpretation [40, 41]. Whether apoE is only needed to initiate the polymerization or also to prepare each peptide for addition to the growing filament is not yet known.

 Even though the key predictions of the polymerization hypothesis, that is apoE serving as an A*β* filament catalyst, have been borne out, the compelling experiments demonstrating that human apoE inhibits filament formation in a mouse background require explanation. Furthermore, data from LaDu and colleagues and by others have shown that lipidated apoE, presumably the prevalent form *in vivo*, binds A*β* with an affinity of E2 > E3 > E4 [61–64]. Finally, the elegant and thorough experiments of Castellano and colleagues show very convincingly that expression of a human apoE4 transgene (in the absence of mouse apoE) leads to a longer half-life, (i.e., slower clearance) of A*β* in the brain interstitial fluid compared to E2 or E3 [46].

 The apoE-A*β* binding studies might be interpreted as support for apoE functioning in A*β* clearance because apoE2, for example, would bind A*β* tightly and could thereby promote its removal from the interstitial fluid via LRP receptors [50, 61–64]. However, an important feature of any catalyst is that it must bind its substrate only tightly enough to convert it to the transition state structure and then release it as the reaction is completed [65, 66]. If a mutation leads to an overly tight substrate binding, then no further reaction can occur. Thus apoE2 could indeed bind A*β* most tightly, and thereby not only prevent apoE4 from binding and promoting A*β* oligo/polymerization, but also prevent the spontaneous polymerization of the peptide.

 The ability of different apoE isoforms to bind A*β* with different strengths can also explain why human apoE isoforms slow amyloid deposition in the presence of the endogenous mouse apoE, for they may bind A*β* more tightly or differently than mouse apoE and slow the catalytic conversion of A*β* into oligomers/polymers in the mouse background.

**Figure 1:**
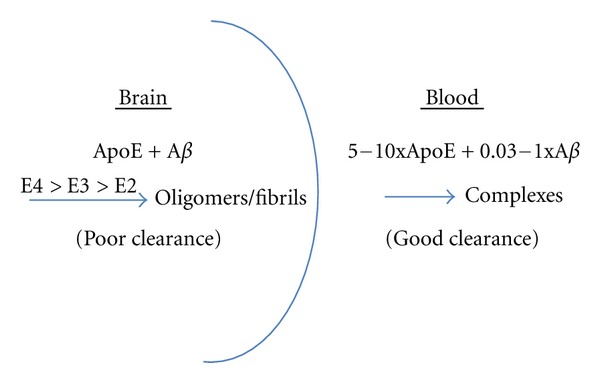
ApoE promotes A*β* Fibril formation in brain and A*β* clearance from the blood. Data from both *in vitro* and *in vivo* experiments indicate that apoE, especially apoE4 promotes the polymerization of A*β* into oligomers and polymers that accumulate in the brain and are difficult to clear. In contrast, the concentration of apoE is higher in the blood, while those of A*β* species are equivalent to or lower than in the brain, promoting the formation and clearance of equimolar apoE-A*β* complexes.

 The data showing that human apoE inhibits A*β* clearance can also be interpreted as reflecting apoE's role in catalyzing A*β* oligo/polymerization. Pathologic macromolecular structures are often resistant to various clearance mechanisms designed for monomeric species, whether by intracellular proteasome degradation or cross-membrane/BBB transfer, thus allowing their accumulation. Only when oligo/polymeric structures are anticipated and physiological clearance mechanisms are in place to handle them, as for antibody-antigen complexes, will clearance be facilitated by conversion to larger structures. Because apoE clearly has the ability to catalyze the conversion of A*β* into oligomeric and polymeric structures, it is reasonable to assume that those structures will be more difficult to clear, and that such difficulty will be detected as clearance inhibition in the brain, for instance, by apoE4, in pulse chase type experiments, while the higher apoE levels in blood may aid the clearance of A*β* from the circulations (Figure 1).

 Finally, the ability of GW3965 and Bexarotene to reduce soluble and insoluble A*β* in the brain of Tg APP mice and improve cognition is most easily understood as resulting from a general activation of the phagocytic activity of microglia. Previous work showed that activation of microglia by acute intracerebral treatment of APP mice with LPS or with Granulocyte-macrophage stimulating factor can similarly reduce amyloid load and improve cognition [67–69] but that long-term peripheral treatment with LPS exacerbated amyloid deposition in an apoE-dependent manner [70]. Stimulation of microglial activity via induction of Toll-like receptor 9 (TLR9) has also been shown to greatly reduce amyloid load and improve cognition [71]. Clearly the interaction of neuroinflammation, microglia, and amyloid load is complex, and the fact that bexarotene “cures” AD in mice is more likely to be despite, rather than because it stimulates expression of apoE. 

## 5. A*β* Binding Proteins and AD Therapy

 A good test of any hypothesis about the pathogenesis of a disease is whether it successfully predicts how the pathogenesis can be inhibited or reversed. For example, small fragments of A*β* corresponding to the amino acid sequences to which ACT (A*β*1-12) and apoE (A*β*12-28) bind can serve as decoy peptides that prevent the binding of apoE to A*β* and its catalysis of A*β* into neurotoxic species [30]. This early *in vitro* work has recently been repeated and confirmed in other laboratories [72, 73]. The decoy principal was extended *in vivo* by preparing a version of A*β*12-28 that has a better plasma 1/2 life and is nonfibrillogenic/nontoxic. It was shown that this peptide could be peripherally introduced into a transgenic APP mouse, where it effectively entered the brain and prevented/reversed oligomer formation, amyloid deposition, and cognitive decline [74–76]. Similarly, amyloid plaques in APP mice contain mouse ACT and injecting A*β*1-11 into one side of the APP mouse brain to block ACT's binding site with endogenous A*β* rapidly reduced amyloid load compared to the other, vehicle-injected side of the brain. Furthermore the inflammatory cytokine IL-1 that is overexpressed in AD brain [18] induces astrocyte expression of ACT [77], and blocking IL-1 expression in APP transgenic mice by Ibuprofen treatment, thereby reducing mouse ACT expression, lowers amyloid formation and restores cognition [78]. Evidently, blocking ACT or apoE expression or function, both *in vitro* or *in vivo*, successfully prevents A*β* pathology and neurotoxicity.

 Apolipoprotein J also binds A*β* and can be shown to aid its passage across the blood brain barrier [79–83]. Interestingly, knocking out either apoJ or apoE reduces amyloid deposition in APP transgenic animals, yet knocking out both leads to robust amyloid deposition at an even earlier age than arises in nonmanipulated APP animals [84]. This result may reflect the ability of mouse ACT to promote amyloid formation, but that in the presence of the stronger binding apoE and apoJ proteins mouse ACT is prevented from exhibiting its catalytic activity.

## 6. Potential Efficacy and Dangers of A*β*-Binding Antibodies as AD Therapy

 The role of apoE and ACT in the Alzheimer pathogenic pathway has potentially general implications. One of the most studied classes of A*β* binding proteins are specific anti-A*β* antibodies, which form the basis of both passive and active immunization therapies for Alzheimer's disease (for review see [85]). The finding that apoE and ACT can catalyze A*β* oligo/polymerization begs the question of whether A*β* antibodies might also promote or inhibit A*β* polymerization. Indeed we found that two A*β* antibodies, 6E10 which is directed to the same the N-terminal sequence bound by ACT, and 13 M, which binds to the C-terminus, function very differently in the *in vitro* A*β* polymerization assay. 6E10 inhibits ACT-catalyzed polymerization of A*β* while 13 M inhibits ACT catalysis much less and even promotes some polymerization itself. Interestingly, the N-terminus of A*β* is also the target of many attempts at AD immunotherapy with the aim of inducing microglial phagocytosis of neurotoxic A*β* species. Yet removing the microglial-binding Fc portion of 3D6 antibodies to A*β*1-5 to generate Fab'2 fragments does not reduce the antibody's ability to remove diffuse amyloid in APP mice [86]. Evidently, only its A*β*-binding feature is required to allow the antibody to remove amyloid. A possible explanation for this result is that the antibody functions by blocking A*β* interaction with mouse ACT. The consequent suppression of ACT-catalyzed oligo/polymerization could thus tilt the dynamic process of plaque development toward depolymerization.

 These results illustrate the fact that A*β*-binding proteins can have multiple effects on polymerization and that their full range of activities must be considered when using them as potential targets or tools for therapeutic intervention.

## 7. Potential Toxic Mechanism of ApoE-Induced A*β* Oligomers

 Although A*β* oligomers have been shown to be highly neurotoxic *in vitro* and *in vivo*, and their formation is promoted by apoE4, the mechanism of their toxicity is still being elucidated. The data reviewed above coupled to other recent findings suggest a novel mechanism for A*β* toxicity that encompasses the essential role of apoE. Specifically, A*β* oligomers bind to and inhibit certain microtubule motors that are essential for the function and stability of the mitotic spindle—Eg5/kinesin5, Kif4A, and MCAK [87]. Similar motors, including kinesin 5, are also present in mature neurons [88, 89]. We have found recently that inhibition of MT motor function by A*β* or by the specific kinesin 5 inhibitor Monastrol prevents the efficient transport of receptors such as the LDLR, the NMDA neurotransmitter receptor, and the p75 neurotrophin receptor to the cell surface, resulting in reduced function ([90]; in preparation). Similarly, apoE, particularly apoE4, has been shown to reduce the cell surface levels and function of NMDA, AMPA, and apoEr2 receptors in neurons [91]. This latter finding can now be understood as potentially reflecting the ability of apoE4 to promote the conversion of endogenous neuronal A*β* into oligomers, which then inhibit MT-based transport of key cellular components such as receptors to their functional location.

## 8. Conclusion

**Figure 2:**
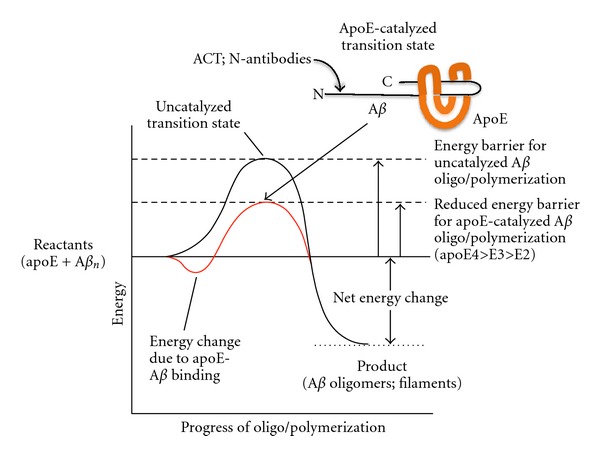
Conceptual energy diagram of ApoE-catalyzed A*β* oligo/polymerization. Although A*β* can polymerize spontaneously, the reaction is greatly promoted by apoE *in vitro* and *in vivo*. This catalysis can be understood in terms of the energy diagram shown. The first energy change, a reduction, occurs as apoE binds to amino acids 12–28 of A*β*, with different apoE isoforms binding with different affinities. Then apoE apparently alters the structure of its bound A*β* to a higher-energy *β*-sheet conformation (the transition state), which allows additional A*β* molecules to add and form a larger oligomer or fibril. These products have lower energy than either the transition state or the initial reactants (apoE and A*β*), thus driving the reaction to completion. Because the energy of the apoE-A*β* transition state is lower than either the transition state of monomeric A*β* in a *β*-sheet conformation, the oligo/polymerization reaction is effectively catalyzed by apoE. ApoE4 evidently forms the lowest energy transition state and thus strongly catalyzes the reaction, apoE3 catalyzes the reaction less well, and apoE2 likely forms such a high energy transition state that it effectively inhibits the spontaneous A*β* polymerization reaction. Antichymotrypsin (ACT), which binds to A*β* amino acids 1–12, also catalyzes A*β* polymerization, while A*β* antibodies can either promote A*β* fibrillization themselves or interfere with ACT or apoE-catalyzed polymerization. Molecules, including antibodies, that prevent apoE or ACT binding to A*β* are being developed as AD therapies that leave the normal physiological functions of A*β* and apoE or ACT intact, while blocking their pathological interaction.

 In sum, it appears that the preponderance of the data can be most consistently interpreted as showing that the brain inflammatory protein apoE plays a catalytic role in the AD/DS amyloid cascade and consequent cognitive decline, with binding and clearance differences between the apoE isoforms reflecting their differing abilities to bind to A*β* and catalyze its conversion into neurotoxic macromolecular species (Figure 2). This conclusion, and the *in vivo* demonstration that blocking apoE-A*β* interaction prevents AD in a mouse model, suggests that this decoy approach should be translatable into human patients and serve as an effective new approach to AD therapy.

Other A*β*-binding proteins may be similarly manipulated by a decoy approach to reduce oligomerization and polymerization of A*β* into neurotoxic species. However, the finding that different antibodies to A*β* can both inhibit ACT-catalyzed A*β* polymerization and promote polymerization of A*β* itself, argues that immunotherapy must be approached with care to avoid the use or induction of antibodies that can catalyze further oligo/polymerization of A*β*, instead of inducing its phagocytosis and removal. Furthermore, human and mouse intracerebral environments may differ in important ways with respect to the pattern and activities of A*β*-binding proteins and may also respond differently to intervention or inflammation. Such differences may explain why so many treatments that were successful in reducing amyloid-dependent cognitive decline in transgenic mice have failed to translate into human AD patients.

 Finally, the ability of A*β* oligomers to inhibit key microtubule motors and prevent the transport of neurotrophin, neurotransmitter, and other receptors to the cell surface may underlie their neuronal toxicity. It is apparently the ApoE-, especially E4-dependent formation of such A*β* oligomers, that constitutes the key catalytic step in the AD pathogenic pathway.
